# Phenolysis for Advanced Shoulder Osteoarthritis: A Case Series of a Novel Ultrasound-Guided Approach to Anterior and Posterior Glenohumeral Articular Nerve Branches

**DOI:** 10.7759/cureus.47890

**Published:** 2023-10-28

**Authors:** Felice Galluccio, Tony Kwun-tung Ng, Mario Fajardo Perez, Ece Yamak Altinpulluk, Murray Taverner

**Affiliations:** 1 Department of Rheumatology and Pain Management, Fisiotech Lab Studio, Firenze, ITA; 2 Department of Pain Medicine, Morphological Madrid Research Center, Madrid, ESP; 3 Department of Pain Medicine, Frankston Pain Management, Melbourne, AUS; 4 Department of Anesthesiology, University of Hong Kong, Hong Kong, HKG; 5 Department of Anesthesia and Intensive Care, Chinese University of Hong Kong, Hong Kong, HKG; 6 Department of Anesthesia and Operating Theatre Services, Tuen Mun Hospital, Hong Kong, HKG; 7 Department of Anesthesia and Pain Medicine, Wan Fang Hospital, Taipei, TWN; 8 Department of Anesthesia, Outcomes Research Consortium, Cleveland, USA; 9 Department of Education and Research, Regional Anesthesia and Pain Medicine, UltraDissection, Madrid, ESP; 10 Department of Anesthesiology Research, Ataturk University Medical School, Erzurum, TUR; 11 Department of Pain Management, Frankston Pain Management, Melbourne, AUS; 12 Department of Perioperative Medicine, Monash University, Melbourne, AUS; 13 Department of Anesthesia and Perioperative Medicine, Monash University, Melbourne, AUS

**Keywords:** denervation, osteoarthritis, phenol, neurolysis, shoulder

## Abstract

Introduction: The shoulder is one of the joints most affected by osteoarthritis, with a prevalence of almost 20% in adults over 65 years of age. Various treatments have been proposed to control osteoarthritis pain, including radiofrequency, pulsed and thermal, and recently cryoanalgesia. We propose in this series of cases a new approach to analgesic therapy with chemical denervation with phenol.

Materials and method: Patients who underwent phenolysis for shoulder osteoarthritis at our institutions in Italy and Australia between August 2022 and May 2023 were included. All patients included in our report provided written consent for publication. This chemical neurolysis technique consisted of two injections. First, the anterior shoulder capsule was denervated by a modified deep SHAC (Shoulder Anterior Capsule) approach to cover the anterior terminal articular branches of the axillary nerve, lateral pectoral nerve, and subscapularis nerve. Second, the posterior shoulder capsule was denervated by a posterior glenoid approach to cover the terminal articular branches of the suprascapular nerve (SSN).

Results: We included a total of 11 patients in this case series. Ten of 11 patients were affected by shoulder osteoarthritis, of which three had rotator cuff tendinopathy and three had full-thickness cuff tears. One patient had chronic subluxation of a shoulder prosthesis. After treatment, all patients significantly reduced pain immediately after treatment and, two weeks later, recovered joint movement and improved quality of life. No adverse events or loss of motor function following treatment.

Conclusion: We presented a novel chemical approach to shoulder denervation, which was shown to be another effective way of improving pain and function in advanced glenohumeral arthritis.

## Introduction

The shoulder joint is one of the most common joints to have osteoarthritis, with a prevalence of 16.1% to 20.1% of adults older than 65 years [1]. It usually refers to the osteoarthritis of the glenohumeral joint, while other joints, such as the acromioclavicular joint, would also contribute to shoulder pain. It is traditionally common to perform radiofrequency of the shoulder nerves for chronic degenerative pain conditions, like pulsed radiofrequency of the suprascapular nerve (SSN) [2,3]. Radiofrequency ablation of this nerve at the suprascapular fossa has also been reported, but the motor function of supraspinatus and infraspinatus would be sacrificed, and this procedure is often insufficient to cover the whole shoulder joint [4].

With recent advances in understanding shoulder innervations, various modified denervation approaches to the shoulder joint, such as cryoablation and thermal radiofrequency, have been developed to handle advanced shoulder osteoarthritis in recent years [5-12]. However, all these approaches require multiple needle punctures, sometimes requiring fluoroscopic guidance and very long procedure times, and are generally very expensive and not so readily available. Peripheral neurolytic blocks represent one of the therapeutic possibilities for patients with pain resistant to usual pharmacological and non-pharmacological therapies [13]. For this reason, we developed a novel chemical denervation approach by phenol, which is readily available and very low cost and requires only two ultrasound-guided injections. This approach consists of a modified deep SHAC (Shoulder Anterior Capsule) block for anterior branch neurolysis and a posterior glenoid approach for suprascapular articular terminal branch neurolysis [14]. This case series examines the analgesic and functional benefits of our novel approach to phenolysis in advanced shoulder osteoarthritis.

## Materials and methods

Patients who underwent phenolysis for shoulder osteoarthritis at our institutions in Italy and Australia between August 2022 and May 2023 were included. All patients included in our report provided written consent for publication. According to our respective local ethics committee, no approval was required. The only exclusion criteria from enrollment were patient refusal, skin infection at the intended needle site, allergy to a chemical neurolytic agent, and an uncorrectable coagulopathy.

In short, this chemical neurolysis technique consisted of two injections. First, the anterior shoulder capsule was denervated by a modified deep SHAC approach [15]. This managed to cover anterior terminal articular branches of the axillary nerve, lateral pectoral nerve, and subscapularis nerve. Second, the posterior shoulder capsule was denervated by a posterior glenoid approach to cover the terminal articular branches of the SSN. 

**Figure 1:**
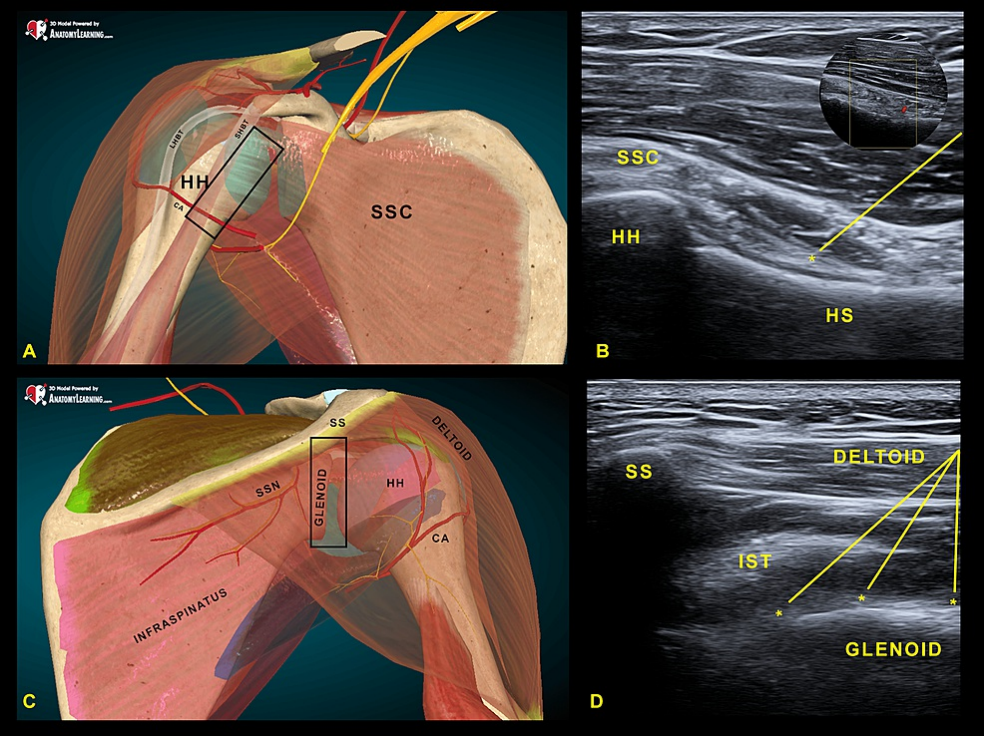
Ultrasound-guided shoulder phenolysis. Anatomical drawing of the anterior (A) and posterior (C) aspects of the shoulder. The black box indicates the position of the ultrasound probe. (B) Ultrasound image of the anterior aspect with the junction between the lesser tuberosity and the humeral shaft beneath the subscapularis muscle. In the upper right box, a Power-Doppler visualization of the anterior circumflex artery is shown. (D) Ultrasound image of the posterior aspect with the visualization of the scapular spine and glenoid. *marks the injection points. Yellow lines indicate the needle trajectory. (A-B) 3D model powered by anatomylearning.com (authorized use). HH, humeral head; HS, humera shaft; SSC, subscapular muscle; LHBT, long head of bicep tendon; SHBT, short head of bicep tendon; CA, circumflex artery; SSN, suprascapular nerve; IST, infraspinatus tendon; SS, scapular spine

To perform the modified SHAC block, the patient was positioned in supine decubitus with the affected arm externally rotated, as tolerated, so that the subscapularis would be pulled out from the glenohumeral joint. This treatment is designed as an outpatient procedure, so there is no need to set up a venous line or monitor the patient. The risks related to the procedure are minimal given that the needle insertion points are in safe areas, away from blood vessels, and the dose of local anesthetics and phenol used is so low as to minimize any risk and make it indistinct from a normal ultrasound-guided injection. A linear probe (6-12 Mhz or similar) was used to localize the myotendinous junction of the subscapularis and then slowly rotated and slid down until the junction between the lesser tubercle of the humeral head and the proximal humeral shaft was visualized (Figure 1A and Figure 1B). Doppler screening of the terminal branch of the anterior circumflex artery, distal to lesser tubercle, was then performed (Figure 1B). The skin was anesthetized with 1% lignocaine and then the needle was inserted from a caudal to cephalad trajectory and advanced until the needle tip was in contact with the articular capsule (Figure 1B). A diagnostic block with 4 mL bupivacaine 0.5% was performed to predict response to chemical neurolysis.

For the posterior glenoid approach, the patient was positioned in lateral decubitus with the affected shoulder up, and the arm in a neutral position. To identify the posterior glenoid, the ultrasound probe was placed to visualize the shoulder in a transverse plane, followed by putting the glenoid in the middle of the screen. Once it was identified, the probe was rotated to the sagittal plane until a longitudinal image that includes the spine of the scapula on the cephalad side and the glenoid, which would appear rounded in shape, was obtained (Figure 1C and Figure 1D). As before, the skin was anesthetized with 1% lignocaine, and the needle was inserted from a caudal to cephalad trajectory to reach four different points: at the lower end, in the middle point, and at the upper end of the posterior glenoid bony surface, and the last to the bony surface of glenoid underneath the spine of the scapula to cover all the terminal branches that depart from the SSN to the glenohumeral joint. As before, we proceed with a diagnostic block with 0.5 mL bupivacaine 0.5% per point.

A positive diagnostic block was defined as more than 50% pain relief on active or passive shoulder flexion at 45° and an improvement in the range of active or passive shoulder flexion, extension, and abduction by more than 50%. If the diagnostic block was positive, phenolysis was arranged in a separate session with the same approaches and needle trajectories. A total of 6% aqueous-based phenol was used as the neurolytic agent, and the same volume was injected into each target. 0.5 mL 1% lignocaine was injected on needle withdrawal after phenol injection.

A numerical rating scale (NRS: 0-10) was used to assess the dynamic pain before and two weeks after the phenolysis. Oxford shoulder score (OSS) was also used to assess the pain and functional aspects of the shoulder specifically. Ranges of motion in flexion, abduction, and external rotation were evaluated with their differences before and after the phenolysis denoted as DROM.

## Results

We included a total of 11 patients in this case series. Patient characteristics and treatment outcomes are shown in Table 1. Ten of 11 patients were affected by shoulder osteoarthritis, of which three had rotator cuff tendinopathy, and three had full-thickness cuff tears. Finally, a patient was suffering from chronic subluxation of a shoulder prosthesis.

**Table 1:** Patient characteristics and tabular results. GHOA, glenohumeral osteoarthritis; FTTRC, full-thickness tear of the rotator cuff; RCT, rotator cuff tear (partial); TSA, total shoulder arthroplasty; OSS, Oxford shoulder score; NRS, numerical rating scale

Patient number	Age and sex	Pathology	OSS before treatment	NRS before procedure	NRS after procedure	NRS 2W after treatment	OSS 2W after treatment	DROM (%)
1	68/M	GHOA+FTTRC	4/48	8/10	1/10	2/10	38/48	50%-55%
2	73/F	GHOA	11/48	8/10	2/10	1/10	41/48	60%-70%
3	75/F	GHOA	8/48	7/10	0/10	1/10	46/48	60%-60%
4	66/M	GHOA+RCT	10/48	9/10	0/10	2/10	38/48	60%-65%
5	62/F	GHOA+RCT	12/48	10/10	0/10	1/10	40/48	45%-65%
6	81/F	GHOA+RCT	8/48	8/10	2/10	2/10	34/48	50%-70%
7	78/F	GHOA	20/48	7/10	0/10	1/10	45/48	70%-70%
8	83/M	GHOA	18/48	8/10	0/10	1/10	46/48	65%-70%
9	65/M	GHOA+FTTRC	5/48	9/10	2/10	2/10	36/48	55%-60%
10	65/F	GHOA+FTTRC	11/48	7/10	2/10	3/10	26/48	40%-50%
11	79/F	TSA subluxation	5/48	10/10	1/10	3/10	35/48	50%-70%

After treatment, all patients experienced significantly reduced pain immediately after treatment and, two weeks later, enjoyed recovered joint movement (expressed in DROM) and improved quality of life as reflected by their OSS.

There were no adverse events nor any loss of motor function, even partial or temporary, following treatment.

Case presentation

Patient 5

**Figure 2:**
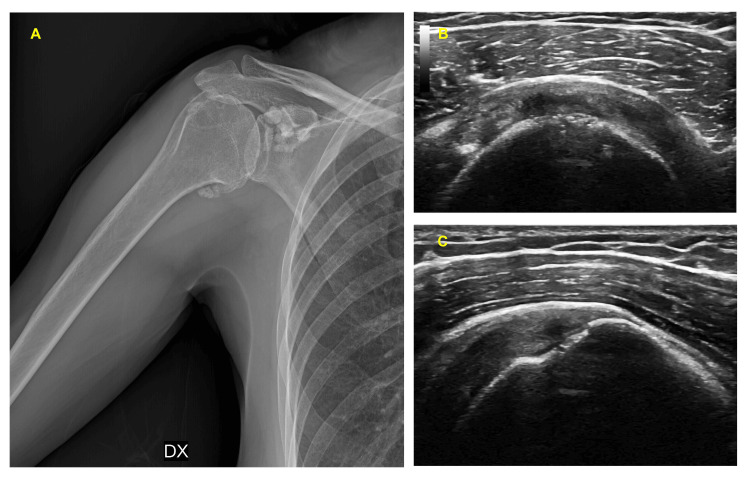
Patient 5. (A) X-ray of the right shoulder of patient 5 with glenohumeral osteoarthritis with gross osteophyte of the lower margin and sub-coracoid and soft tissue peri-diaphyseal calcification of soft tissue. (B-C) Ultrasound of supraspinatus tendon with an intrasubstance partial tear.

A 62-year-old woman suffered from right glenohumeral osteoarthritis with large osteophyte of the lower margin and asymptomatic sub-coracoid and humeral peri-diaphyseal soft tissue calcifications with partial rotator cuff tear and unbearable pain that did not respond to standard treatments and worsened after an accidental fall (Figure 2A,Figure 2B, and Figure 2C). The patient had been treated with opioids and intra-articular infiltrations of methylprednisolone without success. Even physiotherapy treatments did not benefit because limited by pain. Treatment with NSAIDs and radiofrequency was contraindicated for heart disease with PM-ICD implantation and arterial hypertension. Baseline OSS was 12/48, and motion NRS was 10/10. Pre-active range of movement (ROM) was about 10° in external rotation and elevation. The diagnostic block gave a positive result with the disappearance of the pain (NRS: 0/10) and functional recovery (DROM: 45-65%), for which phenolysis was scheduled for the following week. After two weeks, the OSS scored 40/48 and the NRS was 1/10. At the follow-up after four months, the patient maintained the effect and resumed her regular daily activity.

Patient 8

An 83-year-old man suffered from osteoarthritis of the right shoulder, which significantly limited his daily activities and led him to abandon sporting activities. His pain was so intense that it interrupted his sleep at night and forced him to take analgesics daily. He exhausted almost all conservative and less invasive treatment modalities, including intra-articular infiltrations of cortisone and hyaluronic acid, physiotherapy, and physical therapies (TENS and magnetotherapy). He had multiple comorbidities, for instance, hypertensive heart disease, mild renal failure, and chronic gastritis with hiatal hernia. His baseline OSS was 18/48, and the NRS was 8/10. Pre-procedure active ROM was about 20° in external rotation and elevation. He received the diagnostic blocks early in the morning with a rapid response in terms of pain (NRS: 0/10) and functional recovery (DROM: 65-70%), and he received the phenolysis in the afternoon. After two weeks, the OSS scored 46/48 and NRS was 1/10. After some sessions of physiotherapy and home exercises with elastic resistance, he also improved his muscle strength, so much so that he could resume light sporting activity. To date, five months have passed since the phenolysis, and the result of the treatment has remained unchanged for now.

Patient 10

A 65-year-old lady with multiple comorbidities; including bipolar affective disorder, hypothyroidism on thyroxine, rheumatoid arthritis, hypertension, and diabetes mellitus; history of opioid addiction on suboxone therapy; and left hip pain with total joint replacement done. She presented with worsening left shoulder pain after a fall two months ago. Her mental health was relatively stable despite the recent stress from the worsening pain. Her initial OSS was 11/48, and the dynamic NRS was 7/10.

Physical examination revealed generalized left shoulder tenderness with grossly limited ROM in every direction because of pain. The X-ray showed neither fracture nor subluxation. MRI scan revealed advanced osteoarthritis of the glenohumeral joint with full-thickness tear of the supraspinatus and subscapularis.

Management wise, while managing her by a socio-psycho-biomedical approach, we failed conservative treatments like simple analgesics by Panadol and NSAIDS, and hot packs. An off-label trial of topical lignocaine patch also failed. Since she was already on suboxone, further opioid was not considered. After further discussions, we proceeded to the diagnostic block of shoulder articular branches with our novel approach in the pain clinic. She rated a 70% improvement in pain and ROM of 15 minutes after the diagnostic blocks. The effect lasts for a few days.

**Figure 3:**
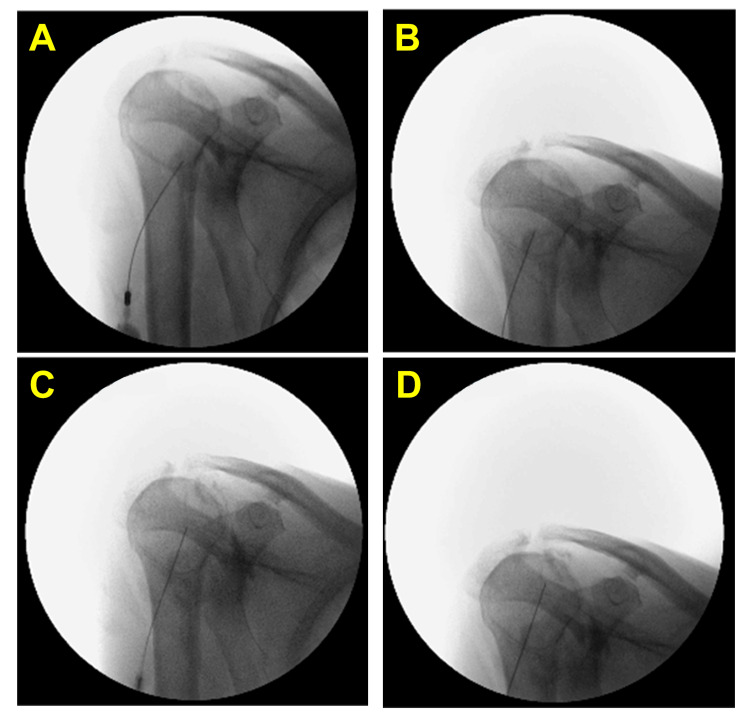
Patient 10. The fluoroscopic images of the posterior glenoid approach of suprascapular nerve articular branch phenolysis with contrast spread. (A) The needle tip on the most caudal part of the posterior glenoid; (B) the needle tip on the middle part of the posterior glenoid; (C) the needle tip on the most cephalad part of posterior glenoid caudal to the spine of the scapula; (D) the needle tip on the posterior glenoid underneath the spine of the scapula.

Phenolysis by the same approach was, therefore, arranged two to three weeks later at the hospital (Figure 3). The NRS was reported as 2/10, 30 minutes after the phenolysis. She, however, experienced intense post-procedural pain on day 2, but it subsided one to two days later. She then rated a 50% reduction in pain with improved ROM two weeks after phenolysis with dynamic NRS of 3/10 and OSS of 26/48. She was subsequently referred to her physiotherapist for mobilization exercises. The pain control and shoulder mobility were maintained three months after the procedure.

Patient 11

**Figure 4:**
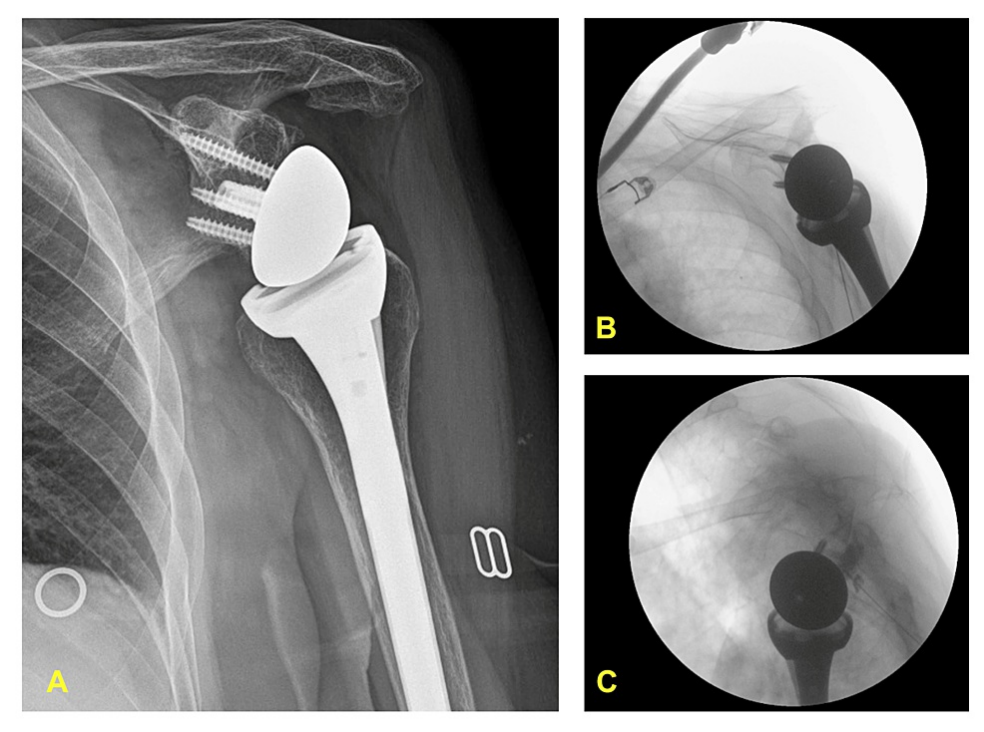
Patient 11. Images of inoperable shoulder subluxation for shoulder phenolysis. (A) The anteroposterior X-ray image revealed a post-replacement glenohumeral joint subluxation. (B) The fluoroscopy image illustrated the final needle position for the deep SHAC phenolysis. (C) The lateral fluoroscopic view showed the final needle position and the contrast spread along the posterior glenoid by the sagittal approach of suprascapular nerve articular branches phenolysis.  The needle trajectory was a caudal-to-cephalad direction.

A 79-year-old lady suffered from severe left shoulder osteoarthritis with a total shoulder replacement done several years ago. However, she still suffered from persistent left shoulder pain, resulting in a frozen shoulder. Her comorbidities included knee osteoarthritis, right radial nerve injury, depression, hypertension, asthma, ischemic heart disease, and psoriasis. It was later found that her prosthetic joint was subluxated (Figure 4A). Nonetheless, no further surgery was offered by her shoulder surgeon. She failed different conservative treatments like potent opioids in the past few years. Her initial OSS was 5/48, and the NRS was 10/10. Her pre-procedural active ROM on flexion, abduction, and extension were only 10°. After the positive result, she then received the diagnostic blocks described above, followed by phenolysis in the same session. For the illustrative purpose, combined fluoroscopy-ultrasound guidance was also used (Figure 4B and Figure 4C). Her shoulder pain and function improved two weeks later with an OSS of 35/48 and dynamic of NRS 3/10. The active ROM was restored to 50-70% of the normal range in flexion, extension, and abduction. The first phenolysis lasted four months before the pain gradually returned to her baseline. A second phenolysis was then performed without another diagnostic block. Her left shoulder has been sustainably improved five months after the second phenolysis.

## Discussion

Shoulder denervation has attracted recent attention in the management of disabling shoulder pain. The pericapsular concept of chemical denervation has recently extended from the hip joint to other joints like the knee, or in acute and chronic pain conditions like cancer or fractures [15-21]. Phenolysis has also been used successfully to treat both adductor and shoulder spasticity, but to date, there are no published studies for the treatment of pain from shoulder osteoarthrosis, with or without rotator cuff partial or full-thickness tears [22,23].

Although the new landmarks for radiofrequency or cryoablation have been advocated lately, they need multiple injections and fluoroscopic assistance [24-25]. They are also expensive and only sometimes covered by health systems or insurance. According to the findings in the SHAC block, we realized that the anteromedial articular branches to the shoulder joint, regardless of their origin, can all be caught in the same place, exploiting their pericapsular path in the terminal section [7]. Hence, the modified deep SHAC approach blocks these terminal sensory nerves without blocking any motor branches to the rotator cuff [14].

Likewise, the posterior glenoid would be the final location for various articular branches of the suprascapular nerve before entering the joint capsule. Hence, denervating the nerves along the posterior glenoid sagittally from the caudal to the cephalad would faithfully cover the posterior sensitive articular innervation.

This chemical denervation approach of shoulder articular branches has the following advantages. First, it can be done under sole ultrasound guidance, also in obese patients, using a curved probe instead of a linear one.

Second, the procedure time is shorter than other ultrasound or fluoroscopy approaches that have been proposed. Third, fewer needle punctures are required than all published techniques; hence, there would be less post-procedural pain. Fourth, because the volume of phenol is small, the procedure may be done in an ambulatory setting so long as there is adequate equipment and monitoring. Finally, the procedure cost is incomparable to that of a radiofrequency or cryoablation, making it accessible to a much larger population.

We are aware of a potential for the neurolytic agent to spread medially from the posterior glenoid, resulting in the involvement of the motor branch of SSN supplying the infraspinatus at the spinoglenoid notch and the medial trunk of the SSN at the suprascapular fossa. Therefore, we inject small aliquots of 0.5 mL phenol at each target point along the posterior glenoid to minimize such a risk, which, as shown in this case series, has never occurred.

This approach can also be performed with other neurolytic agents, such as ethyl alcohol. We chose phenol because in our clinical experience the secondary effects, such as post-procedural pain, are lower than alcohol. The main risks related to the procedure are infection of the needle insertion site, bleeding and bruising, and post-procedural pain. The dosages of phenol used are well below toxicity levels [13]. The greatest risk with neurolytic blocks is linked to accidental intravascular puncture, a rather rare event due to the absence of large vessels in the pericapsular space.

The main limitation of this study is that it is a report of a series of cases, albeit collected in two different centers (Italy and Australia). Nevertheless, this is the first report of the efficacy of phenolysis of the shoulder sensory nerves at the pericapsular level in a range of eligible patients. Further randomized control trial is needed to validate this approach.

## Conclusions

In summary, chronic shoulder pain secondary to advanced glenohumeral osteoarthritis is challenging to treat and often associated with significant functional impairment. While joint denervation by radiofrequency or cryoablation is valid, it may be contraindicated or inaccessible for some patients. We presented a novel chemical approach to shoulder denervation, which was shown to be another effective way of improving pain and function in advanced glenohumeral arthritis. 
